# A cost-effectiveness evaluation of latent tuberculosis infection screening of a migrant population in Malaysia

**DOI:** 10.1038/s41598-023-29648-z

**Published:** 2023-02-10

**Authors:** Erin Barker, Joe Moss, Hayden Holmes, Catherine Bowe, Vinay Suryaprakash, Riccardo Alagna, Vladyslav Nikolayevskyy, Marc Destito, Davide Manissero

**Affiliations:** 1 York Health Economics Consortium, Enterprise House, Innovation Way, University of York, Heslington, York, YO10 5NQ UK; 2 QIAGEN GmbH, 40724 Hilden, Germany; 3 QIAGEN SRL, Via Filippo Sassetti 16, 20124 Milan, Italy; 4 QIAGEN Manchester, Citylabs 2.0 Hathersage Road, Manchester, M130BH UK; 5 QIAGEN AG, Garstligweg 8, 8634 Hombrechtikon, Switzerland

**Keywords:** Tuberculosis, Quality of life

## Abstract

To estimate the costs and benefits of screening for latent tuberculosis infection (LTBI) in a migrant population in Malaysia. An economic model was developed from a Malaysian healthcare perspective to compare QuantiFERON-TB Gold Plus (QuantiFERON) with the tuberculin skin test (TST). A decision tree was used to capture outcomes relating to LTBI screening followed by a Markov model that simulated the lifetime costs and benefits of the patient cohort. The Markov model did not capture the impact of secondary infections. The model included an R shiny interactive interface to allow adaptation to other scenarios and settings. QuantiFERON is both more effective and less costly than TST (dominant). Compared with QuantiFERON, the lifetime risk of developing active TB increases by approximately 40% for TST due to missed LTBI cases during screening (i.e. a higher number of false negative cases for TST). For a migrant population in Malaysia, QuantiFERON is cost-effective when compared with TST. Further research should consider targeted LTBI screening for migrants in Malaysia based on common risk factors.

## Introduction

Tuberculosis (TB) is a serious infectious disease which predominately affects the lungs. Despite progress in reducing TB prevalence and mortality, it remains a global health threat; affecting 10 million people in 2019 and causing 1.4 million deaths^1^. Latent TB infection (LTBI) is a persistent immune response to Mycobacterium tuberculosis and is characterised as asymptomatic and non-infectious^2^. However, without appropriate treatment, approximately 5–15% of LTBI cases develop into active TB infection over a patient’s lifetime^1^. This process is known as reactivation. Reactivation rates have been shown to depend on a variety of risk factors including comorbidities that may weaken the immune system and the amount of time a patient has been infected with LTBI^3,4^. Those who have recently contracted the infection are more likely to develop active TB than those who have carried the infection for many years, meaning the reactivation rate of LTBI decreases over time^4^.

The United Nations Sustainable Development Goals and the World Health Organisation (WHO) End TB Strategy both include targets that aim to end the global TB epidemic by 2030^5^. To this end, detecting and treating LTBI is key to preventing the development of active TB and the spread of the disease^6^.

Systematic LTBI testing and treatments have been shown to be beneficial in scenarios where the likelihood of exposure to TB disease is high, for example, among recent migrants from countries with a high TB burden^7^. The cost-effectiveness of LTBI screening of migrants in low TB burden countries has also been demonstrated by numerous studies^6,8–12^. However, there is less evidence available for countries that are considered a medium TB burden. Malaysia is classified as a medium TB burden country with an estimated 92 cases per 100,000 people^13^. Of all TB cases registered in the Malaysian national TB surveillance database between the years 2014 to 2017, 12.7% of the patients were non-Malaysian^14^. More than 90% of these cases originated from high TB burden countries including the Philippines, Indonesia, Myanmar, Nepal, and Bangladesh^14,15^.

A common diagnostic test for LTBI is the Mantoux test, also known as the tuberculin skin test (TST)^16^. The TST is carried out by injecting intradermal purified protein derivate from Mycobacterium TB cultures into the forearm of an individual. One problem in the clinical application of the TST is its cross-reactivity with antigens present in the Bacillus Calmette-Guérin (BCG) vaccine strain and some non-tuberculous mycobacteria (NTM) causing opportunistic infections. The BCG vaccine is used to protect against TB and is one of the most commonly used vaccines in the world^17^. A reaction to the TST may be as a result of the BCG vaccine thus leading to false-positive results and a decrease in the positive predictive value of the TST^18^. Therefore, alternative diagnostic tools for the detection of LTBI have been explored.

The interferon-gamma release assay (IGRA) is a whole blood assay used to detect LTBI. IGRAs employ stimulation of lymphocytes by two antigens (ESAT-6 and CFP-10) highly specific for Mycobacterium TB complex bacilli but absent in BCG. There are two commonly used IGRAs, the T-SPOT.TB (T-SPOT) test and QuantiFERON-TB Gold Plus (QuantiFERON). The two IGRAs have been shown to have excellent levels of agreement with high sensitivity and specificity^19,20^. A meta-analysis found that both the T-SPOT and QuantiFERON have consistently high specificity, whereas the TST specificity is low and variable in BCG vaccinated population^21^. Hence, IGRAs have been shown to be more accurate indicators of the presence of LTBI than the TST^19^. However, the WHO guidelines and the Malaysian Ministry of Health both state that either the TST or IGRAs can be used for LTBI screening^15,22^.

QuantiFERON works by directly measuring the concentration of interferon-gamma (IFN-γ) in the blood released by lymphocytes upon stimulation with TB specific antigens^16,20^. The test is conducted during a single health care visit by taking a blood sample and testing the blood within a laboratory^23^. In addition to easier testing methods and accuracy, QuantiFERON is unaffected by discrepancies in results from subjective reading since it uses a consistent cut off for positive and negative results^23^.

Current Malaysian guidelines state that LTBI screening and treatment should be considered for recent immigrants (< 2 years) from high TB prevalence countries^15^. While a TST or IGRA can be used for the diagnosis of LTBI, IGRA is not widely available in Malaysia due to cost and laboratory requirements^15^. While the up-front costs of screening may be higher for IGRA testing, the long-term costs and benefits should also be considered when considering screening options.

It is not currently known which of the available LTBI screening tests offers the most cost-effective approach to screen and treat recent migrants from high TB burden countries in Malaysia. Therefore, the aim of this study was to estimate the potential costs-effectiveness of QuantiFERON in comparison with TST for the migrant population in Malaysia. The results of this study aim to support policy makers in Malaysia and in other medium burden TB countries with a large migrant population from high burden countries.

## Methods

### Overview

**Figure 1:**
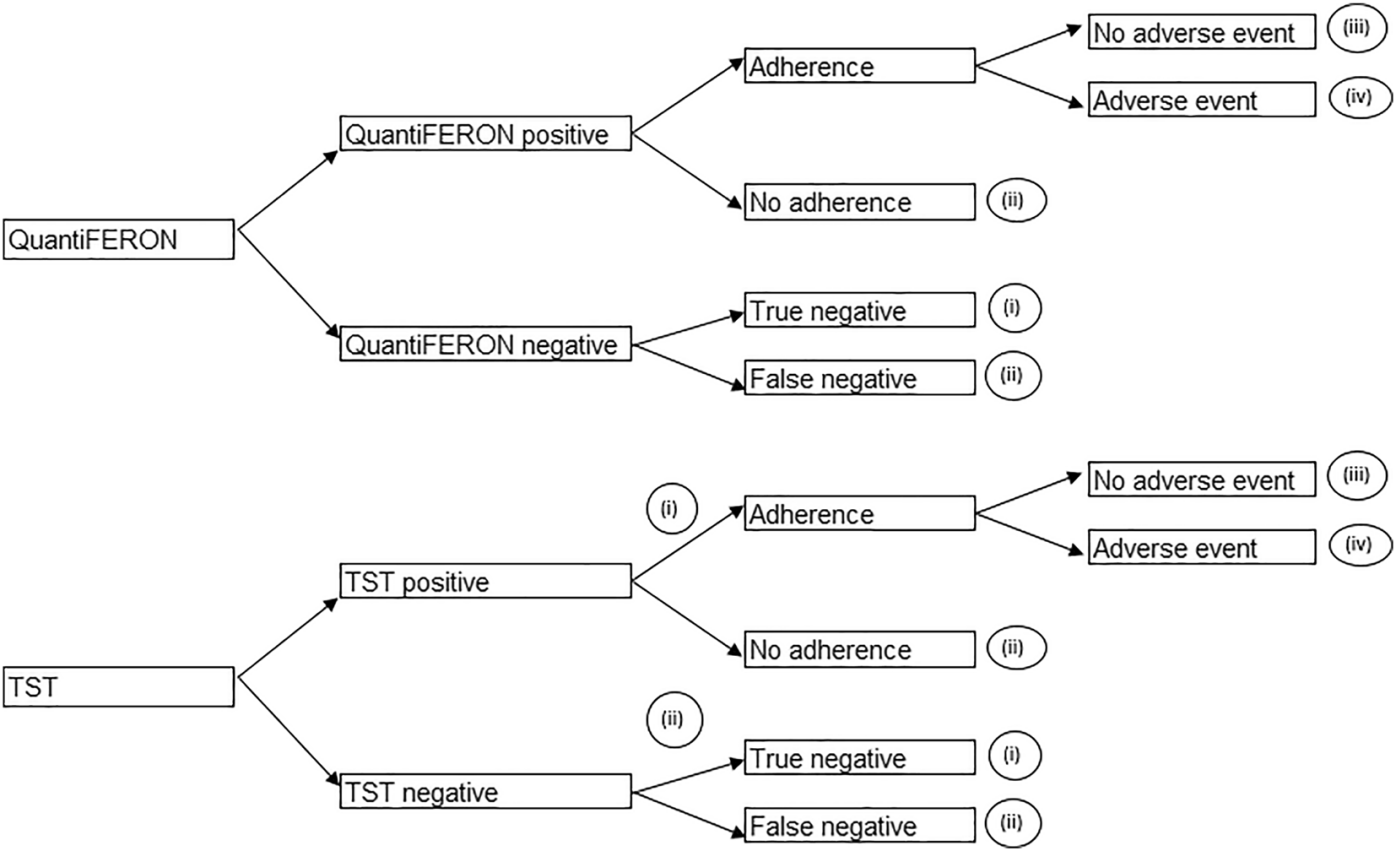
Decision tree structure. The decision tree structure for QuantiFERON-TB Gold Plus (QuantiFERON) and the tuberculin skin test (TST). The four health states leaving the decision tree are (i) Healthy, (ii) Untreated latent tuberculosis infection (LTBI) (treatment Naïve), (iii) LTBI, taking treatment without adverse events and (iv) LTBI, taking treatment with adverse events.

**Figure 2:**
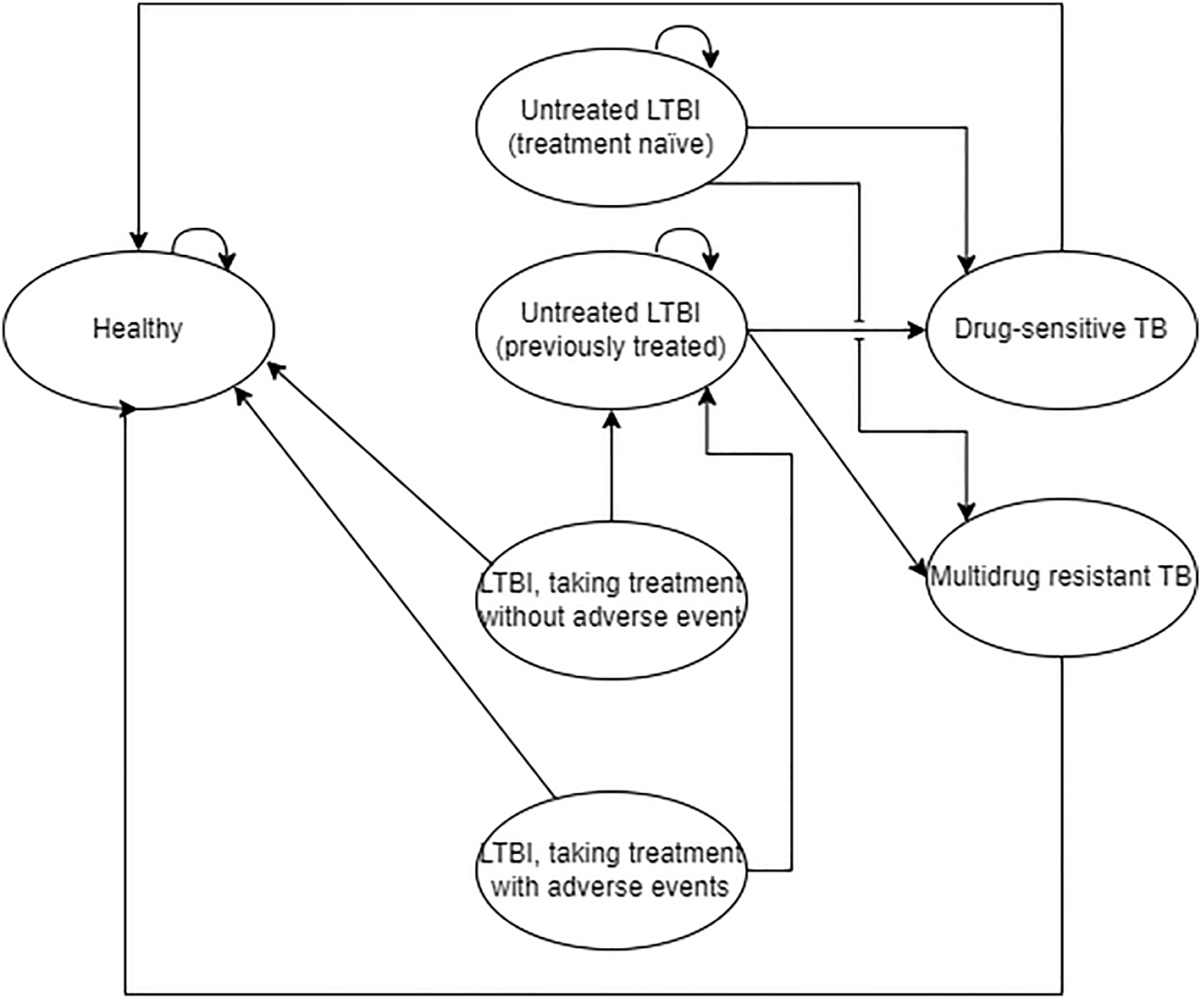
Markov model structure. Markov model structure showing the transitions between seven health states. Transitions into the dead state were permitted from all health states (not shown).

A cohort-based model was developed from a Malaysian healthcare perspective to compare QuantiFERON with the TST. The model composed of two components: a decision tree that captured outcomes relating to LTBI screening (Fig. 1), and a cohort Markov model that simulated the lifetime costs and benefits of the patient cohort (Fig. 2). This study was a modelling study with all data inputs obtained from publicly available sources. Since no patient-level data were used, ethical approval was not required.

Prior to entering the model, it was assumed that the cohort were screened for active TB and that all current active cases were identified as recommended by an advisory board of clinical and health economic experts. Therefore, no active TB cases entered the model at the point of LTBI screening.

Patients entered the model at the decision tree stage. The decision tree starts with the cohort being screened for LTBI with QuantiFERON or TST. A negative result from a screening test indicated either a true negative or a false negative for LTBI. Therefore, a proportion of those with a negative result were true negatives (free of LTBI) whilst the remaining false negatives had undiagnosed LTBI.

Patients with a positive result from a screening test followed the LTBI treatment pathway. However, it was assumed that a proportion of patients diagnosed with LTBI did not adhere/agree to treatment from the point of prescription and therefore remained untreated.

Following the screening stage, patients entered the Markov model. This composed of eight, mutually exclusive, health states with a 1-year cycle length over a lifetime time horizon. These health states were chosen in accordance with previous TB screening models^6,24^ and following advice from an advisory board of clinical and health economic experts. These health states were:

i. Healthy (no LTBI)
ii. Untreated LTBI (treatment Naïve)
iii. LTBI taking treatment without adverse events (AEs)
iv. LTBI taking treatment with AEs
v. Untreated LTBI (previously treated)
vi. Drug sensitive TB (DS TB)
vii. Multidrug resistant TB (MDR TB).
viii. Dead

Therefore, upon leaving the decision tree, patients entered into either the (i) Healthy (ii) Untreated LTBI (treatment Naïve) (iii) LTBI, taking treatment without AEs or (iv) LTBI, taking treatment with AEs state.

Transitions between Markov health states were determined by a set of transition probabilities (Table 1). A patient in the healthy state would always remain in the healthy state as the model was not designed to consider secondary infections or subsequent screening processes. Patients treated for LTBI (with or without AE) either returned to full health or transitioned into the untreated LTBI (previously treated) state. Patients with untreated LTBI (treatment Naïve or previously treated) either remained untreated or transitioned into an active TB state (DS TB or MDR TB). LTBI reactivation curves were used to estimate the reducing probability of LTBI reactivation each cycle. Transitions into the dead state were permitted from all health states. It was assumed that all patients treated for active TB were cured with no further risk of TB reactivation.

**Table 1:** Transition probabilities.

From	To						
	(i) Healthy	(ii) Untreated LTBI (treatment Naïve)	(iii) LTBI, taking treatment without AE (%)	(iv) LTBI, taking treatment with AE (%)	(v) Untreated LTBI (previously treated)	(vi) Drug-sensitive TB being treated	(vii) Multidrug resistant TB being treated
(i) Healthy	100%	0%	0	0	0%	0%	0%
(ii) Untreated LTBI (treatment Naïve)	0%	Residual*	0	0	0%	Calculated from LTBI reactivation rates*	Calculated from LTBI reactivation rates*
(iii) LTBI, taking treatment without AE	Calculated from treatment effectiveness	0%	0	0	Calculated from treatment effectiveness	0%	0%
(iv) LTBI, taking treatment with AE	Calculated from treatment effectiveness	0%	0	0	Calculated from treatment effectiveness	0%	0%
(v) Untreated LTBI (previously treated)	0%	0%	0	0	Residual*	Calculated from LTBI reactivation rates*	Calculated from LTBI reactivation rates*
(vi) Drug-sensitive TB being treated	100%	0%	0	0	0%	0%	0%
(vii) Multidrug resistant TB being treated	100%	0%	0	0	0%	0%	0%

*AE* Adverse event, *LTBI* Latent tuberculosis infection.

*Values were updated every cycle.

### Model inputs

Input parameters on the probabilities of outcomes of screening and management of latent and active TB were obtained from published literature (Table 2).

**Table 2:** Model inputs.

Parameter	Value	Standard error	PSA distribution	References
Population size (migrants)	29,940	N/A	N/A	United Nations^25^ (average annual increase between 2015 and 2019)
Average age	29.2	N/A	N/A	Department of Statistics Malaysia^26^
Proportion male	0.604	N/A	N/A	International Organization for Migration^27^
LTBI prevalence	0.172	0.017	Beta	Yap^28^
All-cause mortality	Age-dependant	N/A	N/A	WHO^29^
DS TB mortality risk	0.07	0.007	Beta	Al Abri et al.^6^
MDR TB mortality risk	0.11	0.011	Beta	Mbuagbaw et al.^30^
Treatment efficacy	0.69	0.069	Beta	International Union Against Tuberculosis^31^
Adherence rate	0.67	0.067	Beta	Al Abri et al.^6^ (assumes 6-month daily isoniazid treatment)
Probability of AE (drug-related hepatotoxicity)	0.02	0.002	Beta	
Initial reactivation rate (treatment naive)	0.0333	1.14	Beta	Gupta et al.^4^
Initial reactivation rate (previously treated)	0.073	0.16	Beta	Derived from Gupta et al.^4^
Proportion of MDR TB cases (treatment naive)	0.012	0.001	Beta	WHO Tuberculosis profile: Malaysia^32^
Proportion of MDR TB cases (previously treated)	0.031	0.003	Beta	
Proportion BCG-vaccinated	0.99	N/A	N/A	Malaysia: WHO and UNICEF estimates of immunization coverage, 2019 revision^33^
Sensitivity of TST	0.77	0.03	Beta	Pai^21^
Specificity of TST (BCG-vaccinated)	0.59	0.07	Beta	
Specificity of TST (non BCG-vaccinated)	0.97	0.03	Beta	
Sensitivity of QuantiFERON	0.91	0.03	Beta	Sotgiu^34^
Specificity of QuantiFERON	0.95	0.01	Beta	
Utility values				
Healthy	1.00	N/A	N/A	Dion et al.^35^ and Guo et al.^36^
Untreated LTBI	1.00	N/A	N/A	
LTBI, taking treatment without AE	0.99	0.099	Beta	
LTBI, taking treatment with AE	0.85	0.085	Beta	
DS TB	0.80	0.08	Beta	
MDR TB	0.58	0.058	Beta	

*AE* Adverse event, *BCG* Bacillus Calmette–Guérin, *DS* Drug sensitive, *LTBI* Latent tuberculosis infection, *MDR* Multi-drug resistant, *PSA* Probabilistic sensitivity analysis, *TB* Tuberculosis, *TST* Tuberculin skin test, *WHO* World Health Organization.

It was assumed that patients with LTBI were treated with 6-month daily isoniazid (INH) as recommended in the 2018 WHO LTBI guidelines^37^. Hence, the base case inputs for adherence, adverse events, and treatment efficacy were based on 6-month INH. The efficacy of treatment was based on patients who completed treatment because patients who did not adhere to treatment were excluded from the “LTBI taking treatment” health states in the cost-effectiveness model. These patients did not incur costs and moved into the untreated (treatment Naïve) health state.

The sensitivity and specificity rates of each screening option were used to determine the proportion of individuals who were correctly identified as having LTBI as well as the proportion of individuals who were misdiagnosed. The specificity of the TST is greatly reduced in individuals who have received the BCG vaccine. Hence, a parameter for the proportion of the cohort who have been vaccinated was included in the model in order to calculate a weighted average of TST specificity.

Quality adjusted life-years (QALYs) were accrued when patients entered the decision tree and continued into the Markov proportion of the model. Each health state was associated with a utility value, representing the average health-related quality of life experienced by patients residing in that state.

A cumulative risk curve^4^ was extrapolated and used to calculate the instantaneous hazard per year of an individual in an untreated LTBI health state moving into an active TB health state. Different curves were applied to those who were treatment Naïve or previously treated. The ratio between treatment Naïve and previously treated was assumed to be the same as the general population. Patients who developed active TB were stratified between two health states: DS TB and MDR TB.

The average age of the patient population and the proportion of males were used to estimate the rate of all-cause mortality. All patients in the model were subject to an age-dependent rate of all-cause mortality. All-cause mortality rates were sourced from Malaysian life tables provided by the WHO Global Health Observatory data repository^38^ and were used to calculate the proportion of patients who transitioned into the dead health state in each cycle of the model. It was assumed that LTBI posed no additional mortality risk compared with the general population. The mortality risk due to active TB was implemented as an absolute risk which was applied instead of the general population all-cause mortality rate (it was assumed to incorporate all-cause mortality risk). The mortality risk associated with an AE was implemented in addition to the defined mortality risk, and was applied only to the “LTBI, taking treatment with AE” health state. However, due to lack of evidence, this input was set to 0% in the base case.

Resource use data (Table 3) were combined with unit costs (Table 4) to determine the total estimated costs of screening and management of latent and active TB. Unit costs and resource use were sourced by QIAGEN from national sources for Malaysia unless otherwise stated. Within the decision tree, each branch was associated with a set of resources which were consumed precisely once. The resource use associated with each Markov health state was estimated per cycle, and patients continued to consume these resources for as long as they spent time in the health state.

**Table 3:** Resource use.

Resource	LTBI without AE	LTBI with AE	Drug-sensitive TB	Multidrug-resistant TB
Healthcare worker contact time				
Nurse time (h)	3	6	0	0
General practitioner time (h)	6	14	0	0
Hospital physician time (h)	0	14	3.7	9.6
TB specialist time	0	0	3.7	9.6
DOT sessions	0	0	90	180
Diagnostic tests				
Smear test of sputum	0	0	6	24
Culture of sputum examination	0	0	2	18
Chest X-ray	0	0	3	18
ESR	0	0	5	18
GeneXpert (PCR test)	0	0	2	2
Laboratory time for diagnosis tests	0	0	9	20
Adverse events				
Liver Function test	0	12	2.8	14.4
Lab time for liver function test	0	24	5.5	28.8
Treatment of drug induced hepatotoxicity	0	1	0.2	0.6
Active TB case contacts				
Contacts per active case	0	0	10	10
Other				
Hospitalisation	0	0	1.6	8.4

All resource use were sourced by QIAGEN from national sources for Malaysia. The resource values in the PSA were assumed to have a standard error of 10% and a Gamma distribution was used.

*AE* Adverse event, *DOT* Directly observed therapy, *ESR* Erythrocyte sedimentation rate, *LTBI* Latent tuberculosis infection, *PCR* Polymerase chain reaction, *PSA* Probabilistic sensitivity analysis, *TB* Tuberculosis.

**Table 4:** Unit costs.

Parameter	Description	Cost (USD)	Source
LTBI treatment	The average cost of LTBI treatment per patient	49.50	GDF^39^
Drug-sensitive TB treatment	The average cost of treating drug-sensitive TB per patient	68.90	GDF^39^
Multi drug resistant TB treatment	The average cost of treating multi drug resistant TB per patient	927.38	GDF^39^
QuantiFERON	The cost of a QuantiFERON test for one patient	20.39	QIAGEN
TST	The cost of a TST for one patient	2.40	QIAGEN
Healthcare worker contact time			
Nurse time	The cost of a nurse per hour	3.60	QIAGEN
Hospital physician time	The cost of a hospital physician per hour	47.97	QIAGEN
General practitioner	The cost of a general practitioner per hour	19.19	QIAGEN
TB specialist time	The cost of a TB specialist per hour	47.97	QIAGEN
DOT sessions	The cost of a DOT session	3.60	QIAGEN
Diagnostic tests			
Smear test of sputum	The cost of a smear tests per patient	0.32	GDF^40^
Culture of sputum examination	The cost of a culture tests per patient	6.70	GDF^40^
Chest X-ray	The cost of a chest X-rays per patient	14.39	QIAGEN
ESR	The cost of a ESRs per patient	14.39	QIAGEN
GeneXpert (PCR test)	The cost of a GeneXpert tests to check for drug sensitivity per patient	9.98	GDF^40^
Laboratory technician time	The cost of a laboratory technician per hour	6.00	QIAGEN
Adverse events			
Liver function test	The number of liver function tests per patient	14.39	QIAGEN
Laboratory technician time	The cost of a laboratory technician per hour	6.00	QIAGEN
Treatment of drug-induced hepatotoxicity	The average cost of treatments per patient	836.66	White et al.^41^
Active TB case contacts			
Contacts per active case	The average cost associated with contacts per active case of TB	9.59	QIAGEN
Other			
Hospitalisation	The average cost of hospitalisation per day	47.97	QIAGEN

*AE* Adverse event, *DOT* Directly observed therapy, *ESR* Erythrocyte sedimentation rate, *GDF* Global Drug Facility, *LTBI* Latent tuberculosis infection, *PCR* Polymerase chain reaction, *PSA* Probabilistic sensitivity analysis, *TB* Tuberculosis, *TST* Tuberculin skin test.

### Economic outcome

Cost-effectiveness was measured using the incremental cost-effectiveness ratio (ICER). As no explicit cost-effectiveness threshold is available, a cost-effectiveness threshold of $7000 USD was used in this analysis, reflecting the estimated threshold for health care interventions in Malaysia^42^. Both costs and benefits were discounted at an annual rate of 3% in line with the guidelines for economic evaluation in Malaysia^43^.

The lifetime rate of developing active TB (DS and MDR) per patient in the entire cohort was presented as well as the incremental difference between TST compared with QuantiFERON. The incremental differences in lifetime TB rates, in addition to the incremental costs, were used to calculate the additional costs associated with treating one active TB case compared with QuantiFERON. The number needed to treat (NNT) to avoid one additional case of active TB using QuantiFERON compared with TST were also presented.

### Model interface

The economic model was built using R version 4.0.2^44^ with an interactive Shiny interface^45^. The interactive interface is a user-friendly format allowing model inputs to be easily changed. As parameters within the model are changed, the model results automatically update. The model was designed to ensure flexibility so that the model can be adapted to reflect different scenarios and settings.

### Sensitivity analysis

First-order uncertainty around the model input parameters was explored using deterministic sensitivity analysis (DSA). For the DSA, the model results were assessed using monetary benefit. Monetary benefit was calculated as the total quality adjusted life-years (QALYs) generated by a screening method multiplied by the cost-effectiveness threshold and then subtracting the total cost for each screening arm. All model input parameters were included within the DSA. For each parameter an upper and lower bound was evaluated. The bounds were defined as a 15% increase and decrease of the estimates used in the base analysis. Probabilistic sensitivity analysis (PSA) was carried out for a deeper exploration of parameter uncertainties. For PSA, a sample of 5,000 iterations was used, with each iteration using a different set of values for the inputs drawn from their respective distributions, to ensure stable results. Model convergence was assessed graphically. The results were presented on a cost-effectiveness plane alongside the cost-effectiveness acceptability curve.

In addition to DSA and PSA, three scenario analyses were performed:

#### Improved treatment adherence rates

In the base case, LTBI treatment adherence was set to 67%. This was based on a 2020 evaluation of QuantiFERON for the screening of LTBI of a migrant population into Oman^6^ and assumed patients were treated with INH for 6 months. However, adherence rates are likely to vary depending on a variety of factors including treatment regimen. To explore the uncertainty around this parameter, a scenario was run where the LTBI treatment adherence rate was increased to 85%^6^.

#### Improved LTBI treatment efficacy

In the base case, LTBI treatment efficacy was set to 69%. This was based on an International Union Against Tuberculosis and Lung Disease trial^31^ and assumed patients were treated with INH for 6 months. However, efficacy is likely to vary depending on a variety of factors including treatment regimen. To explore the uncertainty around this parameter, a scenario was run where the LTBI treatment efficacy was increased to 93%^31^.

#### Increased LTBI prevalence and active TB reactivation rates

LTBI prevalence and TB reactivation rates were expected to be two of the biggest drivers of cost-effectiveness results. These inputs were varied univariately in the DSA but a hypothetical scenario was conducted where both inputs were changed in a multivariate analysis. LTBI prevalence was increased to 34%, and LTBI reactivation rates increased to 6.6% and 1.4% for LTBI treatment Naïve and LTBI previously treated individuals, respectively (values approximately double the base case).

## Results

The base case results per patient are shown in Table 5. Over a lifetime time horizon, QuantiFERON is both more effective and less costly than TST (dominant). Hence, QuantiFERON is cost-effective when compared with TST.

**Table 5:** Base case results per patient.

Strategy	Cost	QALY	Change in costs	Change in QALYs	ICER (cost per QALY gained)
QuantiFERON	$58.70	24.51			
TST	$78.87	24.51	$20.16	− 0.01	Dominated

Compared with QuantiFERON, the lifetime risk of developing active TB increases by approximately 40% for TST. This means that individuals are less likely to develop active TB following QuantiFERON screening compared with TST. This is largely due to missed LTBI cases during screening (i.e. a higher number of false negative cases for TST) resulting in an increase in the number of cases which develop into active TB over a lifetime. The number of additional active TB cases per 100,000 is 170 for TST when compared with QuantiFERON. Finally, compared with QuantiFERON, the number needed to screen to prevent one additional case of active TB for TST is 577. This means that for every 577 people screened, the TST test will result in one additional case of active TB due to a higher number of false negative cases for TST.

### Sensitivity analysis

In the DSA, the biggest driver of the economic results is discount rate for benefits. Plots for the 10 biggest drivers of the economic model based on DSA can be found in Supplementary Fig. S1. The results of the DSA did not change the decision outcome in any of the cases.

**Figure 3:**
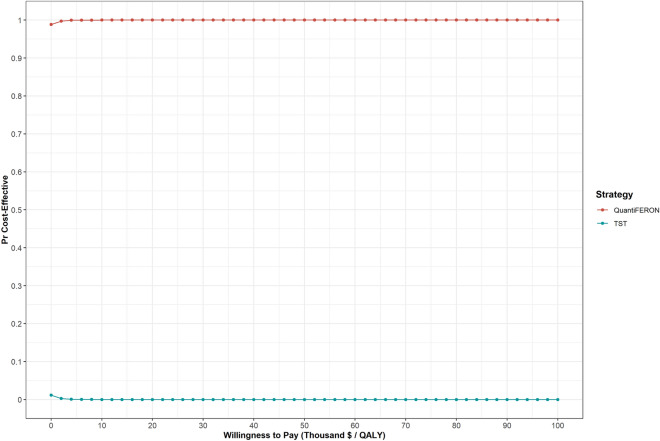
Cost-effectiveness acceptability curves. The results of the probabilistic sensitivity analysis represented as a cost-effectiveness acceptability curves for QuantiFERON-TB Gold Plus (QuantiFERON) versus the tuberculin skin test (TST).

Results from the PSA are shown in Fig. 3. QuantiFERON is cost-effective compared with TST in 100% of the simulations, independent of the willingness to pay threshold chosen (Fig. 3).

Furthermore, in all scenarios examined, QuantiFERON is cost-effective (dominant) compared with TST.

## Discussion

The results of this study suggest that QuantiFERON is cost-effective when compared with TST for the migrant population in Malaysia. In the base case scenario, the probability that QuantiFERON is the most cost-effective option was approximately 100% when compared with TST. While these results should be interpreted with caution given the uncertainties in the model inputs and the number of assumptions made throughout the model, the sensitivity and scenario analyses do indicate that the model results were robust.

Varying the top 10 drivers of the economic model did not change the direction of cost-effectiveness results. However, the DSA highlighted that LTBI prevalence and the reactivation rate of TB (treatment Naïve) have a large impact on the results. This was an important finding and supports the findings of similar studies^46–48^. In this study, LTBI prevalence may be higher than the base case estimate^49^. Based on a meta-analysis, the global prevalence of LTBI was 24.8% when using IGRAs and 21.2% when using TST^50^. However, LTBI prevalence estimates are based on diagnosis tools which are inherently uncertain and depend on a variety of factors, including on the country of origin and immigration patterns^8,47^. Given that it is not possible to know when an individual contracted LTBI through screening methods, the reactivation rate used in this study was estimated by extrapolating reactivation curves and did not consider other risk factors. As such, the accuracy of the reactivation rate used in the model cannot be certain. A hypothetical scenario that investigated increased LTBI prevalence and increased the TB reactivation rate showed QuantiFERON to be cost-effective when compared with TST (dominant).

Due to the lower sensitivity of TST compared with QuantiFERON, there were a higher number of false negatives meaning a larger proportion of the population developed active TB over time, resulting in fewer QALYs. Likewise, due to a lower specificity of TST in a BCG population compared with QuantiFERON, there were a higher number of false positives, which increased the number of patients unnecessarily receiving preventative treatment. This increased the number of patients experiencing treatment related AEs and, again, resulted in fewer QALYs. Furthermore, unnecessary preventative treatment for false positives resulted in higher costs for TST.

The detection and treatment of LTBI is essential in achieving the goal of eliminating TB globally^6^. The WHO recommends targeted screening for LTBI and offering TB preventive therapy among high-risk individuals. This is broadly split into two categories; those who should be systematically tested and treated for LTBI and those where systematically testing and treatment for LTBI may be considered. Migrants from high TB burden countries are included in the group where LBTI screening may be considered^37^. However, LTBI disproportionately affects people living in lower resource settings and the socially vulnerable^51^. As migrants fall into this category^52^, it may be inappropriate to deprive people of screening and TB preventive treatment opportunities without further justification. Thus, no screening was not considered a relevant comparator in this cost-effectiveness analysis.

Other studies have estimated the cost-effectiveness of LTBI screening for migrants with varying results^6,8,12^. A similar study assessed the cost-effectiveness of QuantiFERON versus TST with different preventative treatment regimens for a migrant population in Oman and found that QuantiFERON was dominant when compared with TST across all treatment regimens^6^. Another study evaluated the cost-effectiveness of screening for LTBI using IGRA testing versus no screening in migrants from high TB burden countries to Singapore and found that LTBI screening was only cost-effective when migrants stayed in Singapore for ~ 50 years^8^. However, as previously discussed, no screening may not be considered an appropriate option. Overall, differences in modelling approaches mean that comparisons to other studies should be made with caution^8,12^. The main strength of the model is the flexibility it offers the user. The model inputs can be varied by the user with results updated automatically to allow exploration of different LTBI assays (including other tests endorsed by WHO for example T-SPOT.TB), scenarios (including no screening), and settings. Careful consideration was given to the cost and resource use which is amendable at a granular level. While the model structure cannot be changed by the user, the advice of clinical experts was sought to ensure the model structure reflects real life and WHO recommended practice.

The limitations of the analyses include the lack of available data. There is no national system that collects data for LTBI in Malaysia^53^ or publicly available data on resource use for TB clinical and diagnostic management. Where possible, model inputs were specific to a migrant population in Malaysia. However, in most cases it was not possible to find data for this group, meaning many parameters were obtained from studies performed in other countries and/or in different risk groups. This may have significant implications on the prevalence of TB, as there are many risk factors for TB, including diabetes, alcohol, malnutrition, smoking, and pollution^54^. It is not known whether these risk factors will be found in similar proportions of people from neighbouring countries, meaning the overall risk for TB may be different. Some costs were estimated where no official source was identified.

In the model, treatment for LTBI was assumed to be 6 months of INH. However, there are other LTBI treatment regimens recommended by WHO and the CDC^22,55^. The effect of including alternative treatments is complex to model because treatment regimen will affect parameters such as adherence, the risk of AE, treatment effectiveness, and costs/resources of LTBI treatment. Alternative treatments were not considered in this study but the flexibility of the model allows the user to explore different treatment regimens by varying the associated model inputs.

Another limitation of the model is that is does not capture secondary infections. While there is a cost associated with finding contacts of active TB infections included within the model, the model does not simulate the costs and benefits associated with these secondary infections. This is likely to underestimate the total cost, especially costs associated with active TB cases. The underestimation of costs due to secondary infection in this study is likely to result in a conservative estimate of the cost-effectiveness of QuantiFERON compared with TST due to the higher rate of lifetime active TB cases occurring when using TST. If secondary infections were considered, this would disproportionately increase the costs for TST relative to QuantiFERON, resulting in a lower ICER. Furthermore, any reduction in the number of active TB cases due to screening and treatment for LTBI will result in lower transmission rates (i.e. secondary infections) which is considered a positive externality.

For simplicity, it was assumed that all cases of active TB are cured by treatment within one model cycle. The probability of being cured, if TB treatment is fully adhered to, is high for DS TB but this is likely reduced for MDR TB^34,56^. As a result, the model may underestimate the resource use and costs associated with treatment of MDR-TB. However, this underestimation is likely to result in a conservative estimate of the cost-effectiveness of QuantiFERON compared with TST due to lower rates of active TB for QuantiFERON.

Despite the limitations of the model, the sensitivity analysis and scenarios explored showed the robustness of the model and found no changes in the decision outcomes. Thus, the model is robust to the assumptions and the limitations outlined.

## Conclusion

In conclusion, this model, which was built to estimate the cost-effectiveness of LTBI screening for a migrant population in Malaysia, has shown that QuantiFERON is cost-effective when compared with TST. Further research should consider targeted LTBI screening for migrants to Malaysia based on common risk factors.

## Supplementary Information

Supplementary Information.

## Data Availability

No datasets were generated or analysed during the current study. Data inputs were obtained from published studies or publicly available sources and are reported within this article.
