# Supplementary material for: A cost-effectiveness evaluation of latent tuberculosis infection screening of a migrant population in Malaysia
Source: Sci Rep. 2023 Feb 10;13:2390. doi: 10.1038/s41598-023-29648-z (PMC9918505; doi:10.1038/s41598-023-29648-z)

Figure S1: Results of deterministic sensitivity analysis (DSA)

The 10 biggest drivers of the economic model based on DSA.


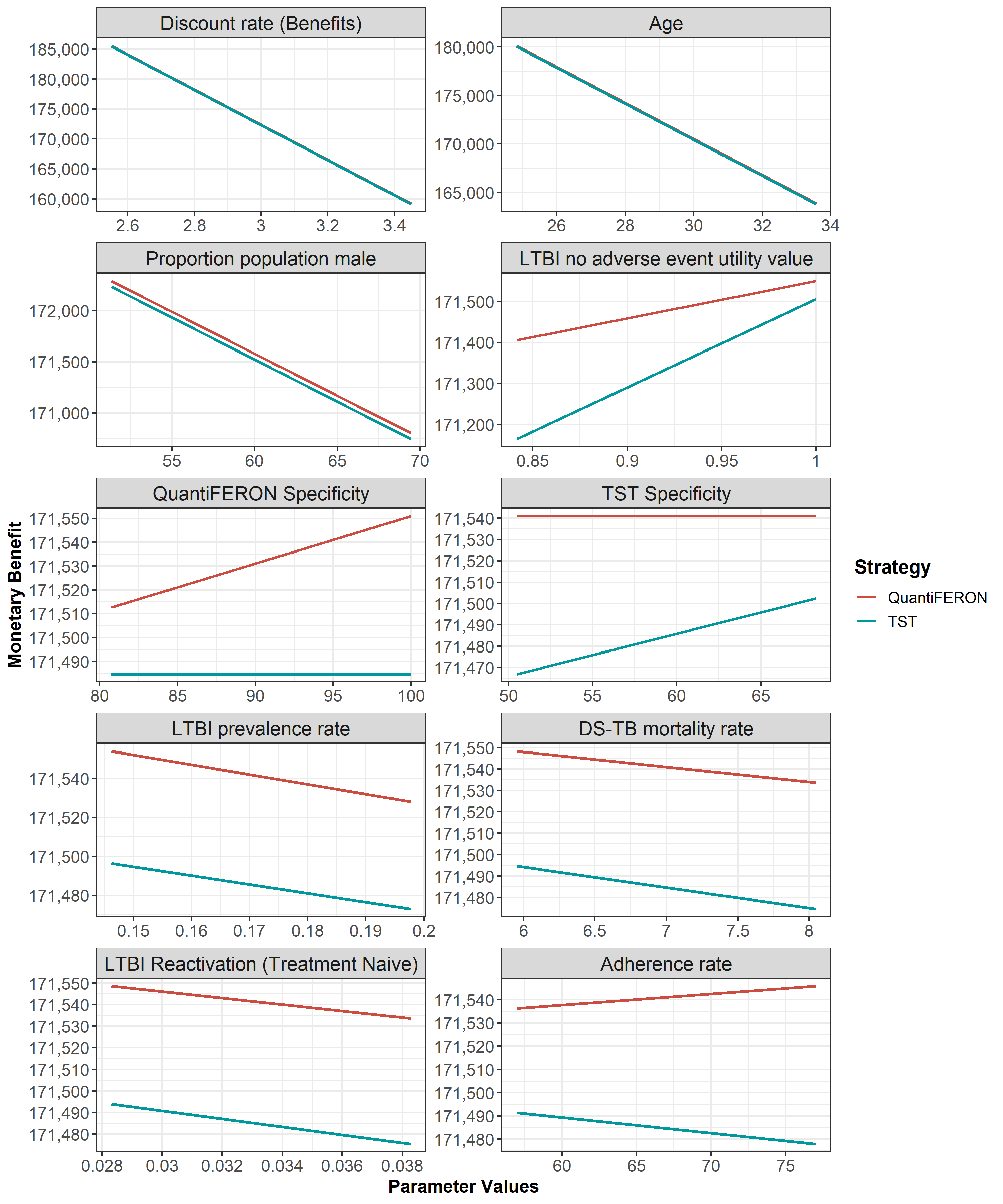

Supplement: Supplementary file 1 — Supplementary Information. [file 41598_2023_29648_MOESM1_ESM.docx]
